# Enhancing Fertility: A Case Report of the Frozen Platelet-Rich Plasma Therapy for Thin Endometrium and Poor Ovarian Reserve

**DOI:** 10.7759/cureus.59271

**Published:** 2024-04-29

**Authors:** Sanket Mahajan, Namrata Choudhary, Jarul Shrivastava, Neha Nawale, Akash More

**Affiliations:** 1 Clinical Embryology, Datta Meghe Institute of Higher Education and Research, Wardha, IND

**Keywords:** intra-cytoplasmic sperm injection, endometrium thickness, ovum pick-up, infertility, platelet rich plasma

## Abstract

A 33-year-old female patient was assessed for primary infertility due to thin endometrium and poor ovarian reserve (POR). The effectiveness of platelet-rich plasma (PRP) therapy was evaluated in terms of thickening the endometrium and enhancing implantation. The patient also had a history of four intrauterine inseminations and one intracytoplasmic sperm injection (ICSI), along with low anti-Müllerian hormone (AMH) and high follicle-stimulating hormone levels which showed POR. Gonadotropins are given to enhance follicular growth, while agonists and antagonists are given to prevent premature luteinizing hormone surge and suppress the top axis. During the first oocyte pick-up (OPU), five oocytes were retrieved. ICSI was done to make fertilization easier. On day 5, the embryos had degraded from their initial high quality. The patient was advised to undergo treatment with PRP. The endometrial thickness was significantly thicker, raising the chance of implantation. The second OPU was scheduled, resulting in the retrieval of 14 oocytes on the same day ICSI was performed. High-quality blastocysts (4AA) were produced and transferred during embryo transfer, and the patient tolerated the procedure well. The clinical success of the pregnancy outcome was confirmed by another beta-human chorionic gonadotropin test.

## Introduction

Throughout the world, infertility affects 17.5% of couples [1]. While in-vitro fertilization (IVF) and other assisted reproductive technologies could help with this issue in certain situations, poor ovarian responder (POR) women frequently have unsuccessful IVF [1]. The most common cause of infertility was POR [2]. Women's ovarian follicle reserves and, consequently, the quality of their oocytes decreases with age. Especially older women generally had an inadequate ovarian reserve [2]. The primary indications for POR diagnosis were age, follicle-stimulating hormone (FSH), and anti-Müllerian hormone (AMH). Nowadays, POR is treated clinically with dehydroepiandrosterone (DHEA), coenzyme Q10, platelet-rich plasma (PRP) injection, acupuncture, and moxibustion. The coenzyme Q10 helps to protect oocytes from reactive oxygen species (ROS) which prevents apoptosis, improves mitochondrial performance, and increases ovarian reserve [3]. Significant therapeutic potential existed for acupuncture and moxibustion treatment to improve POR, as indicated by improvements in POR women's hormone levels and ovarian function repair [3].

A thin endometrium was defined as having a thickness of less than 7 mm on the day of ovulation, the day of human beta chorionic gonadotrophin (β-hCG) injection in newly initiated IVF cycles, or the day of progesterone initiation in frozen-thawed embryo transfer (FET) cycles [4]. The PRP was made from the fresh whole blood of a patient. Red blood cells, which have regenerative and anti-inflammatory qualities, were centrifuged to produce from the process of PRP [5]. Although frozen PRP storage has been reported anecdotally, growth factor concentrations in frozen-thawed PRP have been measured [6].

The PRP therapy was an inexpensive, non-invasive technique. It was composed of high-platelet-concentration plasma from peripherally drawn blood. Plasma contains proteins, hormones, and cytokines and stimulates the growth, division, and proliferation of cells [1,7].

## Case presentation

Patient information

A patient visited a Test Tube Baby Centre located in Mumbai, India, where it was determined that a 33-year-old female patient had primary infertility. The couple refrained from smoking, drinking, using tobacco, or engaging in any other addictions.

Medical/surgical history

The patient had POR and a previous history of four intrauterine insemination (IUI) procedures. There was no medical history of either partner having asthma, heart issues, tuberculosis, or hypertension. The couple's family history was negative, and they had no past history of mental or psychiatric illness. For the first time, they came to our hospital for IVF treatment.

Physical examination/investigation

The male had a body mass index (BMI) of 23.6 kg/m^2^, while the female had a BMI of 22.5 kg/m^2^. According to the husband's semen analysis, morphological defect was 94%, motility was 64%, and sperm count was 40 mil/mL. His semen had a normal morphology of 6%. His report stated that his semen profile was normozoospermic. Table 1 shows the semen reports of the patient observed during the semen analysis procedure.

**Table 1:** Semen parameter of the male partner

Parameter	Observed limit	Reference limit (WHO 2021)
Semen volume	1.8 mL	>1.4 mL [8]
Morphological defects	94%	96% [8]
Normal morphology	6%	>4% [8]
Vitality	47%	>54% [8]
Progressive motility	30%	>30% [8]
Count	40 mil/mL	16 mil/mL [8]
pH	7.2	>7.2 [8]
Color	Opaque white	Opaque white [8]
Viscosity	Liquified	

The female partner underwent an ultrasonography and showed POR. For a thin endometrium (5 mm), we suggested hysteroscopy. Her hormonal levels showed abnormalities with an AMH level of 0.78 ng/mL (normal level: 1 ng/mL to 3 ng/mL) and an FSH level of 14 IU/mL. Table 2 shows the hormonal profile of the female partner.

**Table 2:** Hormonal investigations of the female partner AMH: anti-Müllerian hormone; FSH: follicle-stimulating hormone; LH: luteinizing hormone

Hormonal profile	Patient value	Reference value
AMH	0.78 ng/mL	0.8-1.0 ng/mL [9]
FSH	14 IU/L	10 IU/L [9]
LH	8.0 U/L	5 U/L [10]

Treatment

The gonadotropin-releasing hormone agonists (GnRH) and gonadotropin-releasing hormone antagonists (GnRH antagonists) were the medicines that helped to control the timing and ovulation and encouraged the growth of several follicles in the ovary. A trigger was given to her for oocyte pick-up (OPU). After 36 hours of triggering, OPU was scheduled. During OPU, five oocytes were retrieved (two at the metaphase II (MII) stage, two at the metaphase I (MI) stage, and one at the germinal vesicle (GV) stage). On the same day, intra-cytoplasmic sperm injection (ICSI) was performed. Good-quality cleavage stages were formed, but up to day 5, the embryos had degenerated.

**Figure 1:**
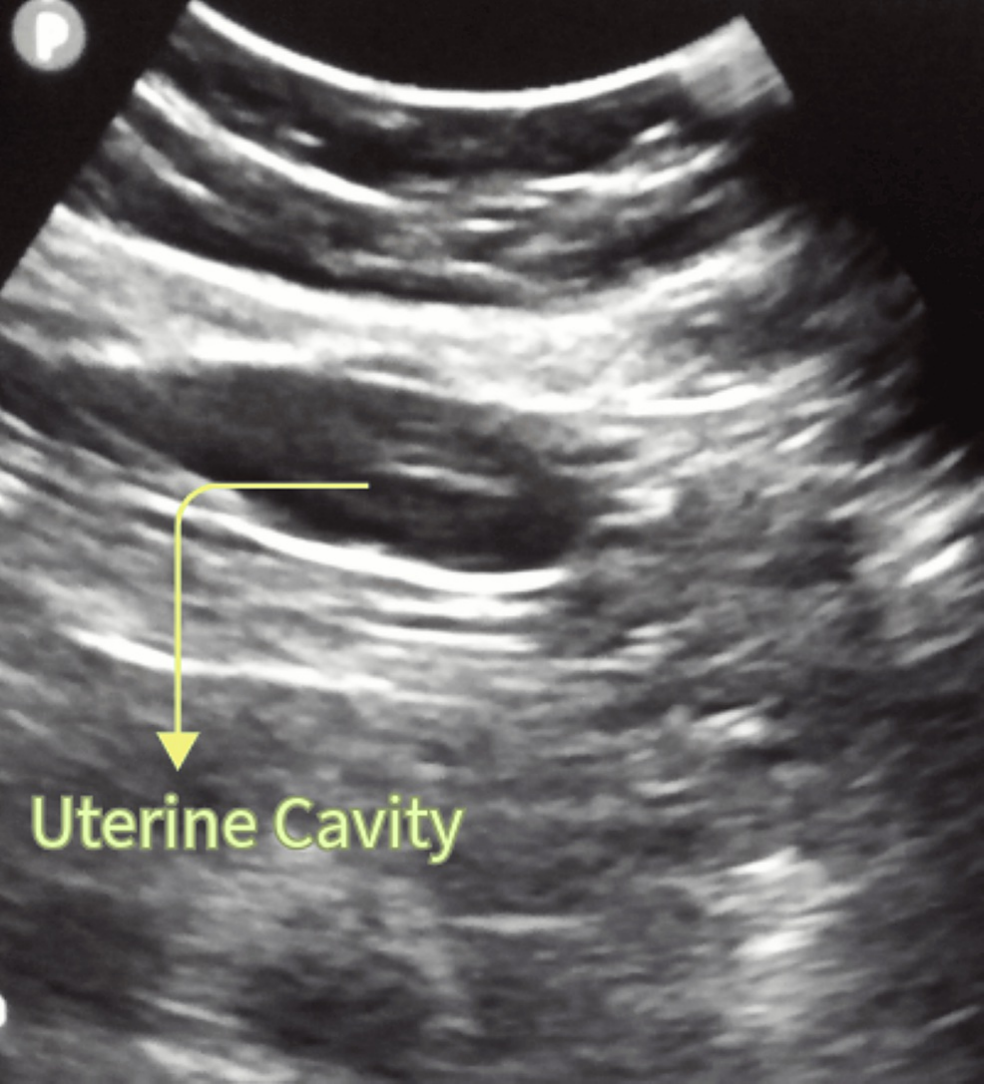
A TVS of the patient's uterine cavity and endometrium on day 17 of the menstrual cycle TVS: transvaginal sonography

After one month, we advised PRP treatment for the thin endometrium. We took two 15 mL test tubes and added 1.5 mL of anticoagulant to each test tube. We collected 13.5 mL of blood from the woman using scalp vein set number 18. A ratio of 1 mL of anticoagulant to 9 mL of blood was maintained. The tube caps were tightly closed and mixed well by inverting them several times. Then we centrifuged at 1500 revolutions per minute (RPM) for 15 minutes. After centrifugation, we separated plasma-rich platelets, ensuring at least 50% volume of plasma without the buffy coat on the red blood count (RBC). Next, we transferred the plasma to another test tube using a sterile pipette without picking up the RBCs. The plasma was then collected in a tube. We gave a second spin of the centrifuge for 15 minutes at 3500 RPM to obtain a platelet pellet. The upper plasma layer obtained was extremely transparent. Using a sterile pipette, the remaining plasma was disposed of, leaving 1 mL on top of the pellet. After the PRP was put in a box, it was frozen in liquid nitrogen for a minute. It was rolled in the palm to melt, completing all three cycles. Platelets were disrupted and released growth factors. We gave a third cycle of centrifuge for 10 minutes at 3500 RPM to remove the platelet membrane debris, or alternatively, we left it as is. Then we added 0.5 mL of released PRP through the intrauterine into the uterus using an IUI cannula. The remaining 0.5 mL of PRP was stored in a freezer compartment for use after three days. We scheduled PRP treatment for the female's first PRP. We administered 0.5 mL of PRP after two days when the sonography report showed the endometrium thickness increased to 6 mm. Then the second PRP was added, increasing the remaining 0.5 mL of PRP, and the endometrium thickness reached 7.5 mm, as shown in Figure 1, which was good for implantation.

The patient was suggested for OPU. We started medication and triggered OPU. After 36 hours, OPU was scheduled. During the procedure, 14 oocytes were retrieved (seven at the MII stage, five at the MI stage, and two at the GV stage). On the same day, ICSI was performed. The male was advised for a fresh semen collection. After collection, we processed the sample, and good-quality sperm and oocytes were used for ICSI. Later, we advised for embryo transfer. After one day, five good-quality 4AA-grade embryos were transferred.

The standard protocols were followed during the IVF process which included controlled ovarian stimulation, obtaining oocytes, and culturing embryos. Positive results were observed in clinical monitoring after the embryo transfer. The process of transferring two excellent embryos into the patient's uterus proved successful.

Follow up

After the embryo transfer, the patient was instructed to resume regular medication. After 14 days of embryo transfer, the serum β-hCG level in a female blood sample tested was 1340 mIU/mL. After she conceived, we counseled the patient to follow up with clinics on a regular basis.

## Discussion

In this study, women with low ovarian reserve were given an intrauterine PRP injection to see whether it impacted them. According to our research, the PRP significantly affected the thickness of the endometrium in women with low ovarian reserves.

Chang et al. were the first to describe the effectiveness of PRP intrauterine injection for endometrial improvement in women with thin endometrium. A total of five women with insufficient endometrium who did not respond well to conventional therapy during the FET cycle received PRP infusions in that trial. All women reported proper treatment responses, and four reported normal pregnancies [11,12].

Multiple studies have shown that the PRP treatment reduces postoperative blood loss, infection, inflammation, and medication needs. Also, the PRP contributes to stimulating osteogenesis and healing wounds and soft tissues [12]. Similar research on the PRP injections sub-endometrically improved clinical pregnancy rates for thin endometrium patients [13]. In a pilot investigation, Zadehmodarres et al. included 10 individuals with a history of insufficient endometrial development. The PRP was infused into patients before the FET. Following PRP and FET, endometrial thickness rose in every patient. There were five young patients. This study suggests that the PRP helped patients with thin endometrium in developing their endometrium [14,15].

Maleki-Hajiagha et al. performed a systematic review and meta-analysis on the PRP, and the results included seven trials with a total of 625 patients (311 cases and 314 controls). When comparing women who received the PRP to controls, the likelihood of chemical pregnancy, clinical pregnancy, and implantation rates increased dramatically (p < 0.001). Regarding miscarriages, there was no difference between the two groups. Endometrial thickness rose in PRP-receiving women after the intervention, whereas it did not rise in the control group. According to the results of this systematic review, the PRP may be used as an alternative course of treatment for individuals with recurrent implantation failure (RIF) and a thin endometrium [16].

The numerous biological studies examining the impact of each PRP variable on the healing potential of platelet concentrates demonstrate the growing awareness of the necessity for PRP standardization. The PRP storage and the effects of freezing and thawing PRP are two of these factors that continue to be disputed. While some researchers steer clear of freeze/thawing due to concerns about potential negative impacts on platelet function and GF release, others view it as a process that physically activates platelets [17]. In this study, increases in endometrium thickness were observed after a cycle of PRP infusions in patients.

## Conclusions

This case study provided help to a patient with a thin endometrium. The implantation process was successful due to the application of PRP, as evidenced by a β-hCG test. To validate the findings of this study, we advised conducting additional research with a larger sample size, as this article was based on only one patient.
